# A Case of Cortical Deafness due to Bilateral Heschl Gyrus Infarct

**DOI:** 10.1155/2017/6816748

**Published:** 2017-03-14

**Authors:** Santhosh Narayanan, K. Abdul Majeed, Gomathy Subramaniam, Arathi Narayanan, K. M. Navaf

**Affiliations:** ^1^Department of Medicine, Government Medical College, Kozhikode, Kerala, India; ^2^Department of Radiodiagnosis, Government Medical College, Kozhikode, Kerala, India

## Abstract

We report the case of a 58-year-old male who presented with an episode of seizure and abrupt onset hearing loss. Neuroimaging revealed acute infarcts in bilateral Heschl gyri. Objective tests of peripheral auditory function were essentially normal and a diagnosis of cortical deafness was made.

## 1. Introduction

Deafness due to cortical origin is an extremely rare entity due to bilateral and wider representation of auditory cortex. It occurs due to damage to bilateral auditory cortices. Clinically recognizing cortical deafness and differentiating it from peripheral auditory problems is a diagnostic challenge as patients respond inconsistently [1]. Advances in audiologic and radiodiagnostic modalities have helped in the realization and better understanding of cortical deafness over the years.

## 2. Case Report

A 58-year-old male, a shopkeeper from Calicut, with past history of diabetes mellitus and ischemic heart disease for 12 years' duration was admitted with a new onset seizure episode. After recovery from postictal state, bystanders noticed that he had difficulty in hearing. They felt he was speaking abnormally, repeating his own words and also without any stimulus. There was no history of fever, headache, vomiting, cranial nerve dysfunction, weakness of limbs, or sensory abnormalities. On examination he was conscious but gave inappropriate response to commands. His vision was normal. He followed written commands, repeating written words or sentences. He could recognize his relatives. Cranial nerves and sensorimotor system were within normal limits. There were no signs of meningeal irritation.

**Figure 1:**
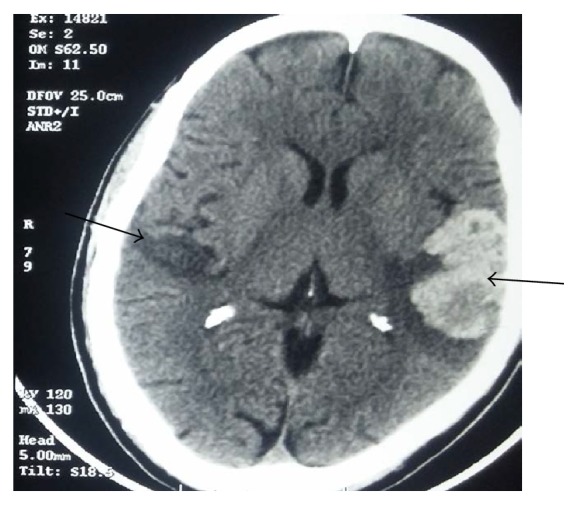
CT head showing infarcts in bilateral temporal lobes with haemorrhagic transformation on left.

**Figure 2:**
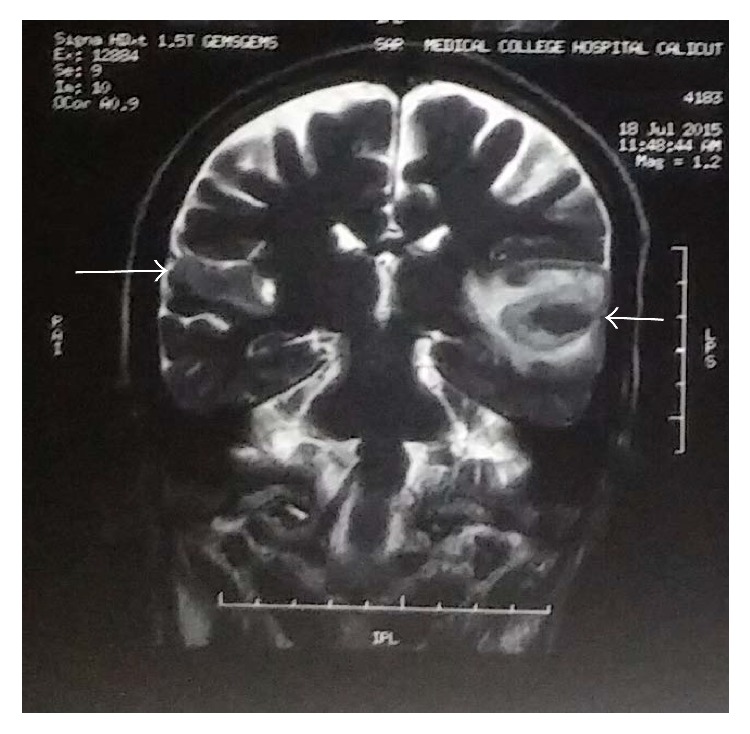
MRI brain showing infarcts in bilateral Heschl gyri.

**Figure 3:**
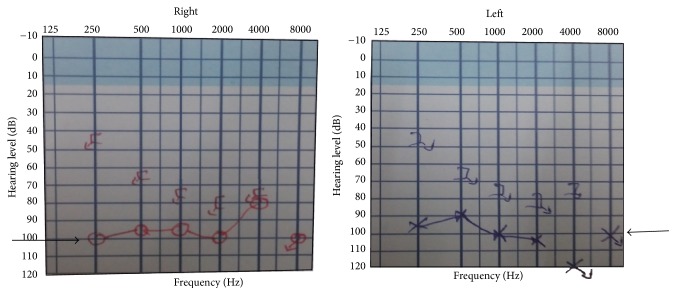
Pure tone audiometry showing bilateral sensorineural hearing loss.

Blood routine investigations showed normal hemogram. Random blood sugar was 326 mg/dl with an HbA1C of 11.1 g/dl. Serum creatinine was 1.3 mg/dl. Electrocardiogram showed normal sinus rhythm with ischemic changes in the anterior leads. He was subsequently seen by an ENT surgeon whose assessment showed normal auricles and tympanic membranes. He did not respond to tuning fork tests. His pure tone audiometry showed bilateral profound sensorineural hearing loss (in Figure 3). Computed tomography scan of brain revealed bilateral acute infarcts in both temporal lobes with haemorrhagic transformation on the left side (Figure 1). MRI brain confirmed bilateral acute infarcts in Heschl gyrus of both temporal lobes with haemorrhagic transformation on left (Figure 2). MR Venogram was normal. We then proceeded with brainstem evoked response audiometry, which showed normal response confirming that brainstem was intact. Middle latency response test (MLR) showed decreased amplitude in Pa wave with increased latency. In the late latency response test (LLR), amplitude was reduced in the P1-N1-P2 complex with increased latency. A diagnostic otoacoustic emission test was done to test the function of outer hair cells which was also normal. Echocardiography revealed mild LV dysfunction with no evidence of any intracardiac thrombus. He was treated with antiedema measures, antiepileptics. On follow-up, his hearing deficit persists but he is able to communicate with others with the aid of speech therapy.

## 3. Discussion

Cortical deafness is a rare disorder due to uniqueness of the auditory pathway. The vestibulocochlear nerve as part of central projections passes through intermediate stations, the cochlear nuclei, superior olivary complex, and inferior colliculus. It eventually reaches thalamus (medial geniculate body) and from there it is relayed to cortex. Primary auditory cortex lies in Heschl gyri, on posterosuperior aspect of temporal lobe deep within the Sylvian fissure, that is, areas 41 and 42. Primary auditory cortex is interconnected with secondary auditory complex or frontotemporal system. Primary cortex is needed to identify basic characteristics of sound, that is, pitch and rhythm. Secondary cortex helps us to distinguish sound as music, speech, or noise. Uniqueness of central auditory pathways is that each of the ears projects to both hemispheres [2].

Deafness of central auditory deficits can be cortical deafness, auditory agnosia, or verbal auditory agnosia. Bilateral temporoparietal lesions (Heschl gyrus, areas 41, 42) can cause cortical deafness and language impairment [3]. But when there is lesion in subcortical structures like projection fibres from medial geniculate bodies or colliculi to auditory cortices, there are deafness and dysarthria. Cortical deafness is severe and rare form of deafness whereby patient is unresponsive to all types of sounds. Auditory agnosia means inability to recognize sounds, different musical notes, or words. This is seen due to defects in secondary auditory cortex area 22 and part of area 21 [4]. Here there is no effect on the perception of sounds and pure tones. Word deafness or auditory verbal agnosia is failure to decode acoustic signals of speech and convert them into understandable words. It is mainly due to left temporal lobe pathology. Such patients can speak, read, and write but they cannot repeat words said to them or write to dictation [5]. Lesions in the midbrain can also cause cortical deafness by interruption of auditory pathways [6]. Cerebral vasospasm following subarachnoid haemorrhage has been reported to cause reversible cortical deafness [7]. Sudden deafness has also arisen as a manifestation of a right temporoparietooccipital AVM [8]. Setzen et al. reported two cases of cortical deafness due to Moyamoya disease [9]. Speech therapy is the only modality which offers benefit to patients with cortical deafness. Goal of speech therapy is to improve comprehension of words and phrases and recognition of environmental sounds.

## 4. Conclusion

Cortical deafness is an unusual disorder due to bilateral representation of auditory pathways. Stroke is the commonest cause. Clinicians should be aware about cortical deafness and should be able to differentiate from other central auditory disorders, as the latter has a better prognosis. Speech therapy improves comprehension and helps in better appraisal of environmental stimuli.
